# Supplementary figures and images for: DaHuangWan targets EGF signaling to inhibit the proliferation of hepatoma cells
Source: PLoS One. 2020 Apr 16;15(4):e0231466. doi: 10.1371/journal.pone.0231466 (PMC7161984; doi:10.1371/journal.pone.0231466)

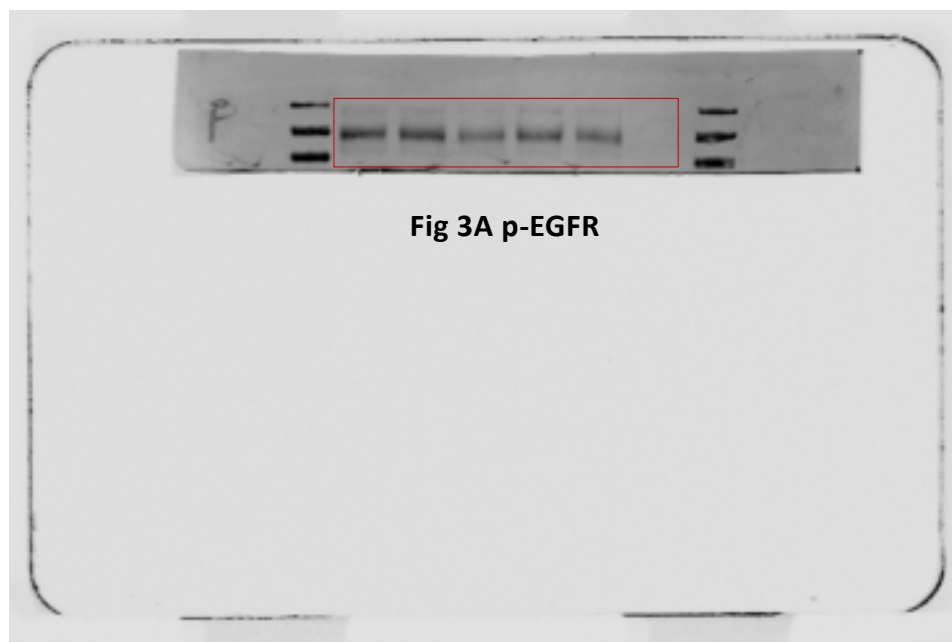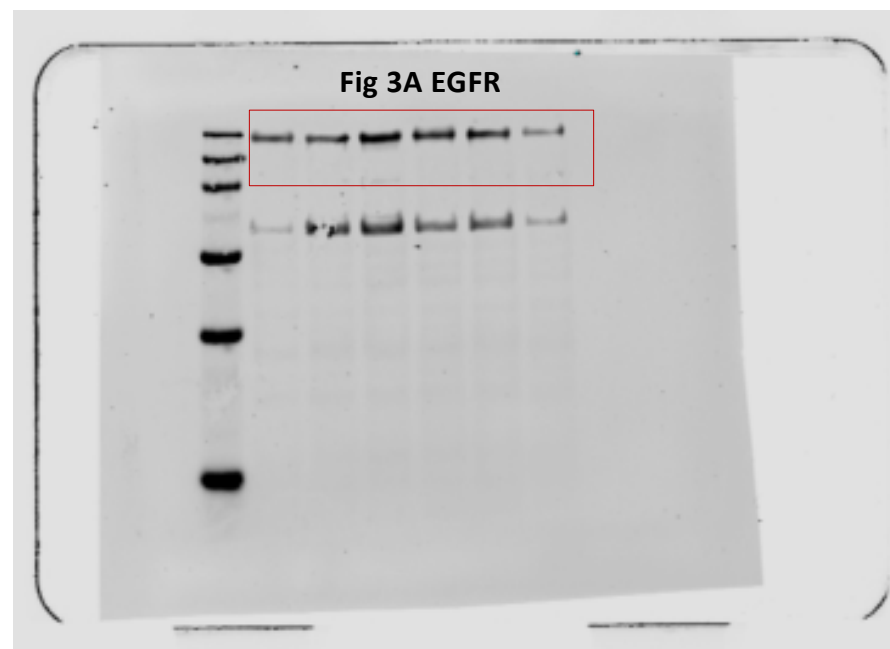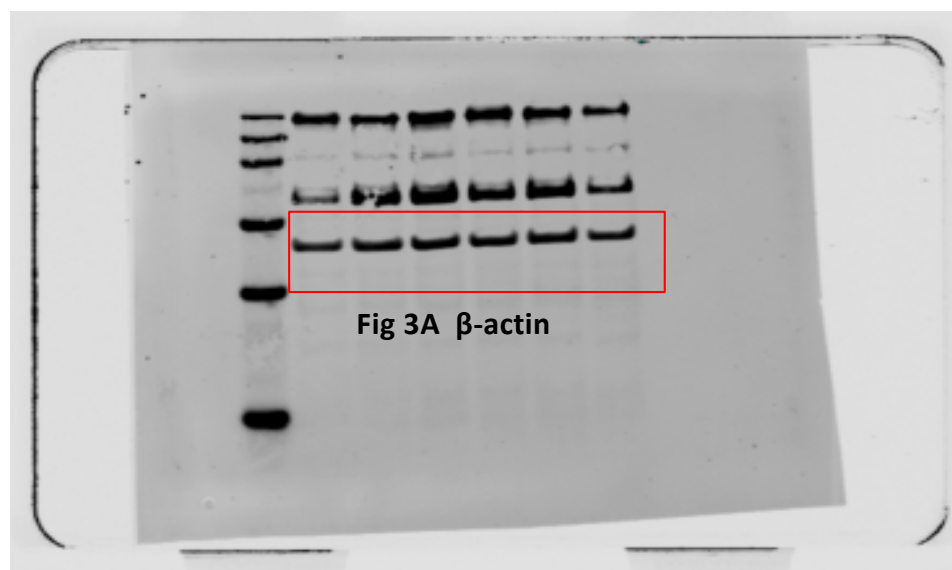

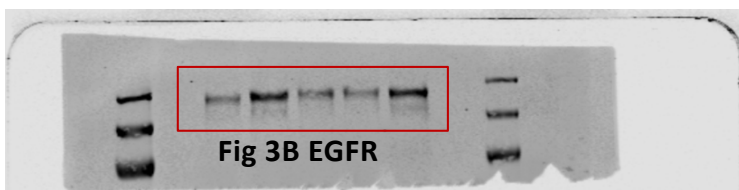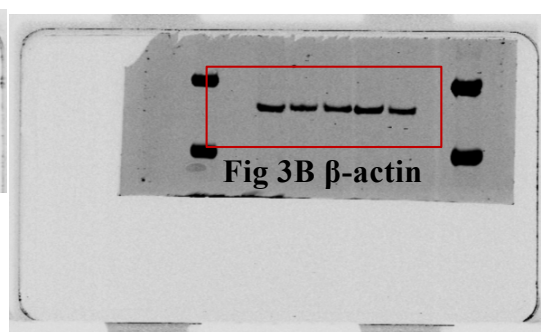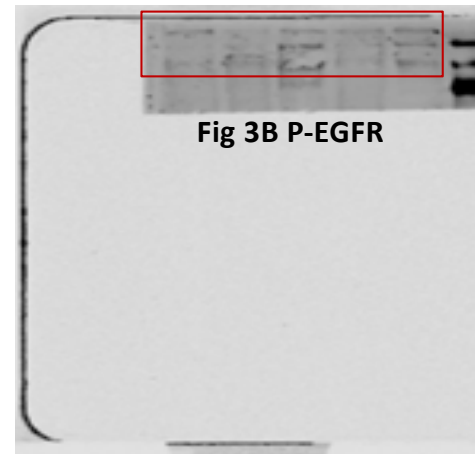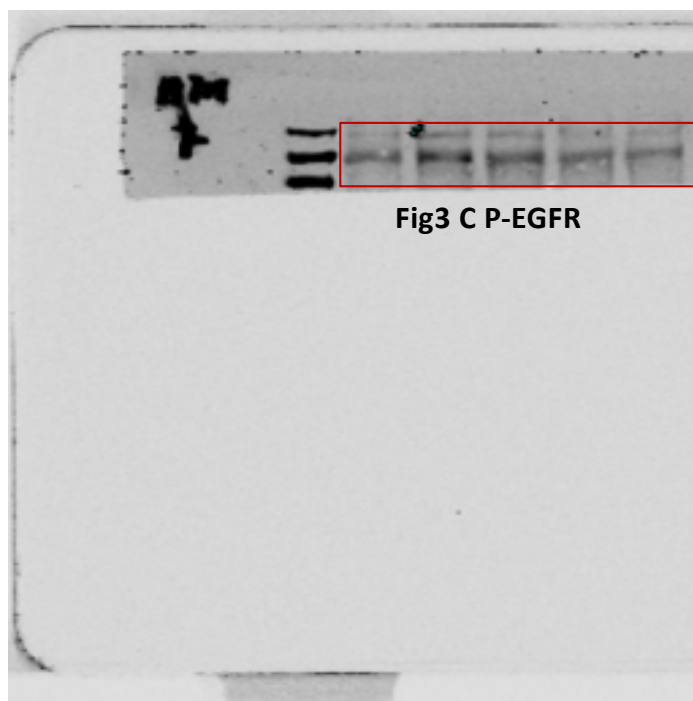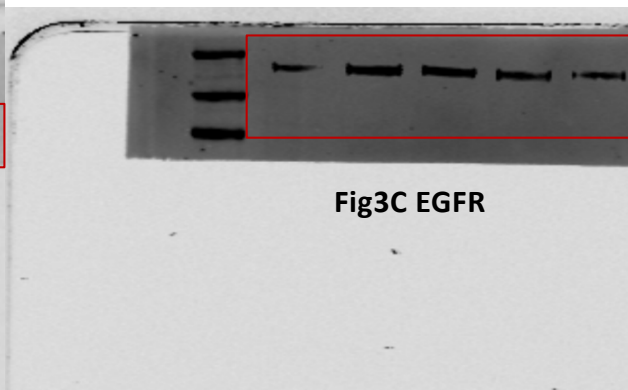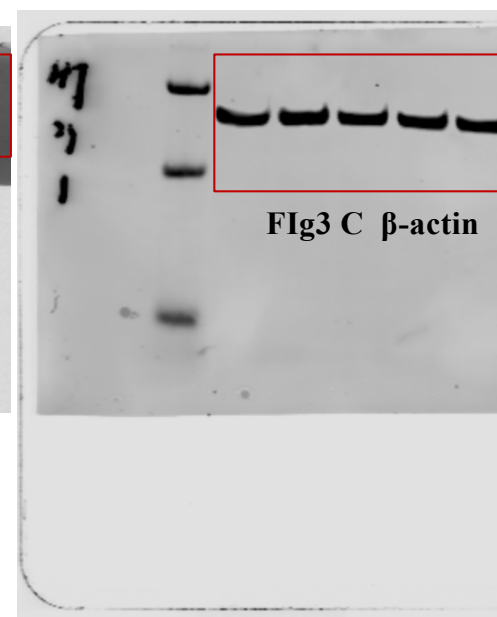

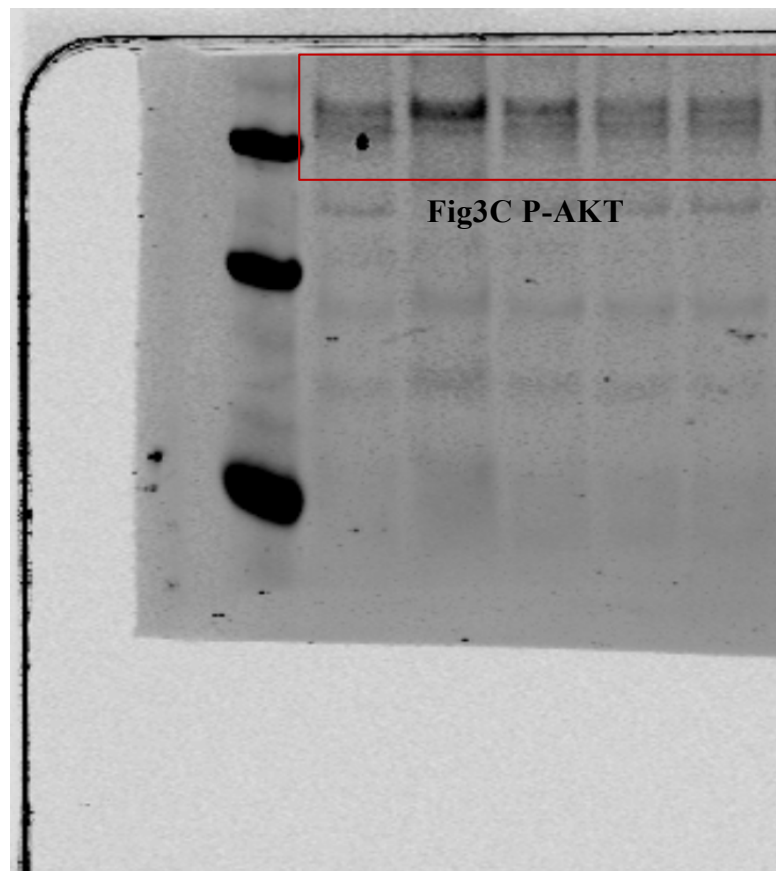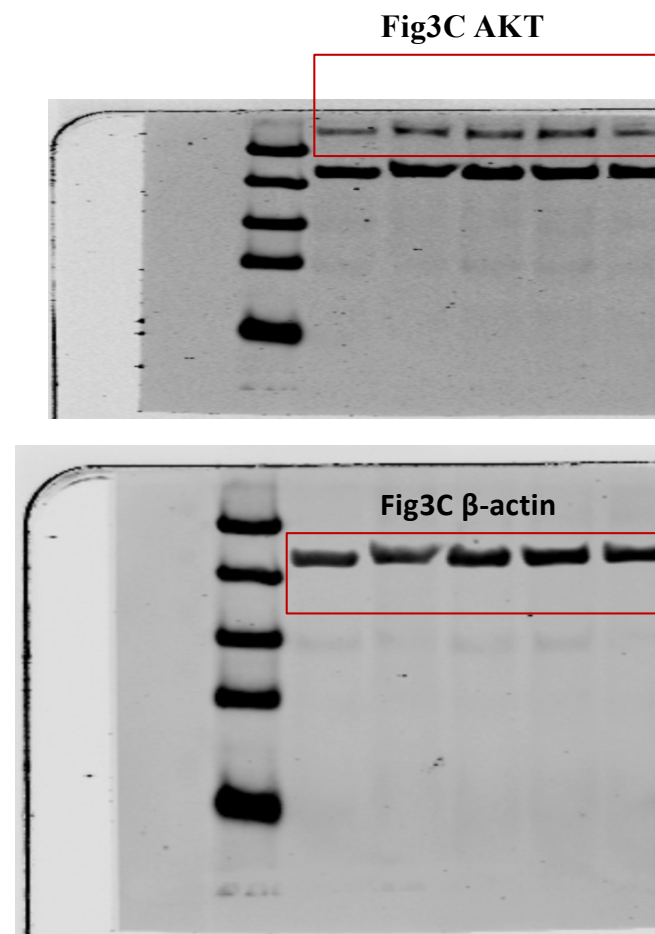

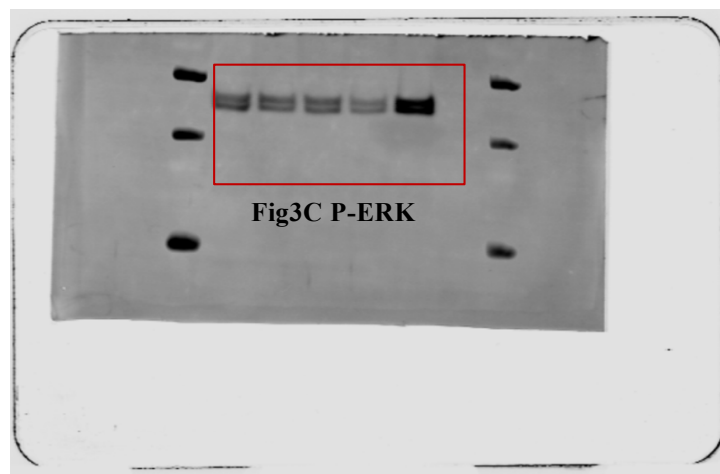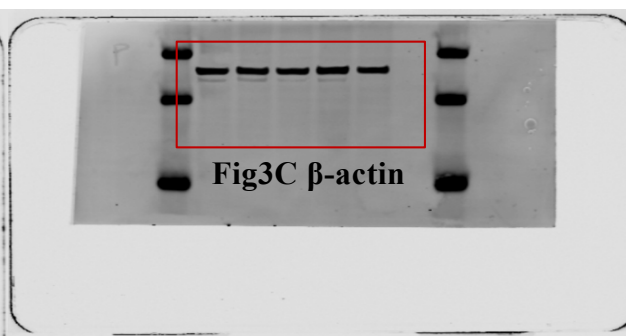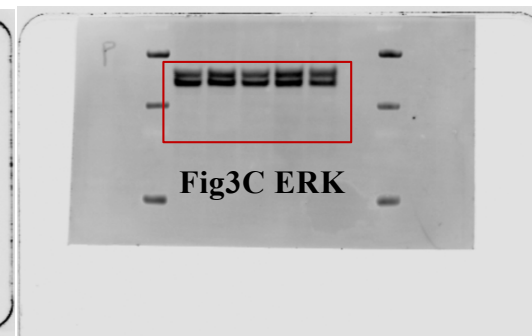

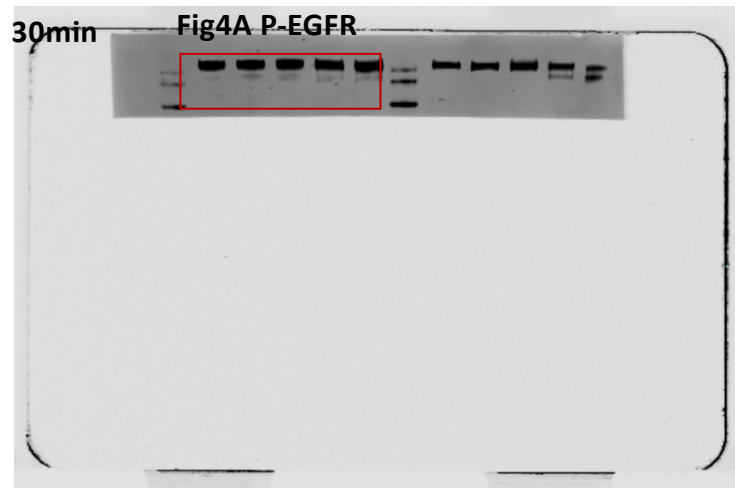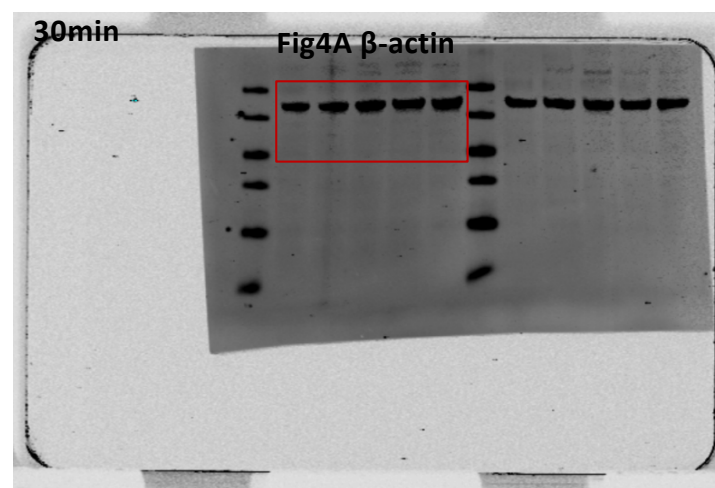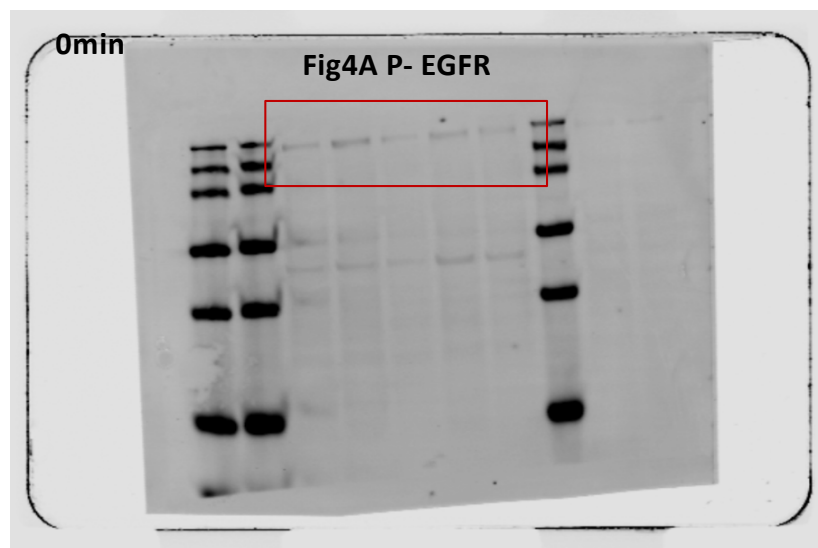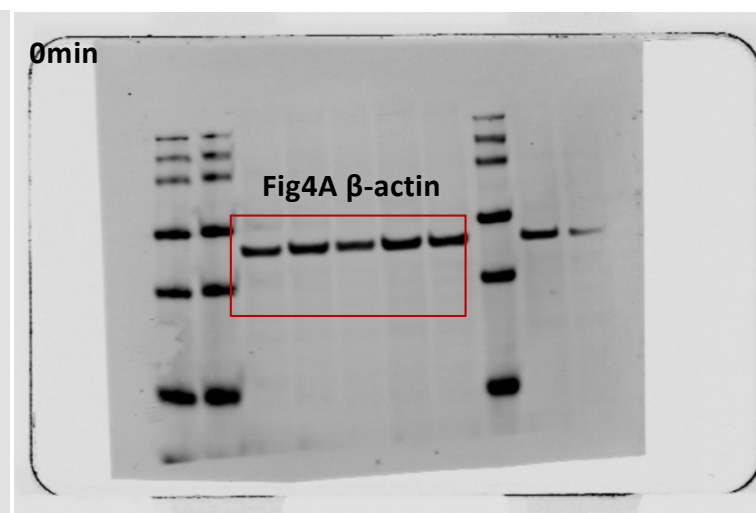

### Cell Surface Protein

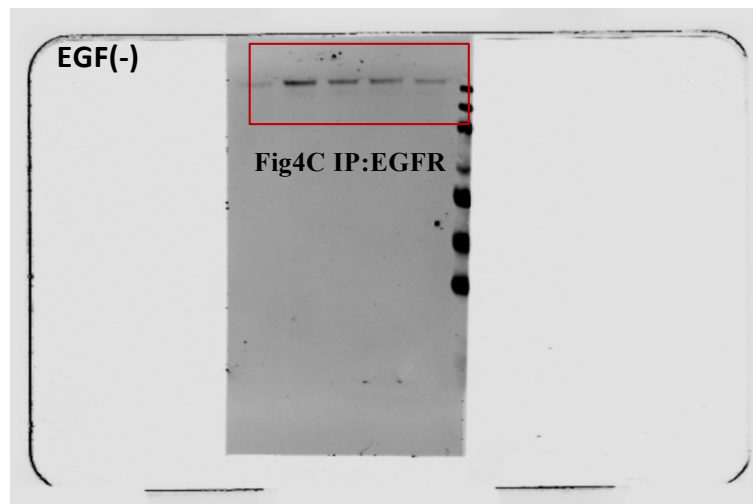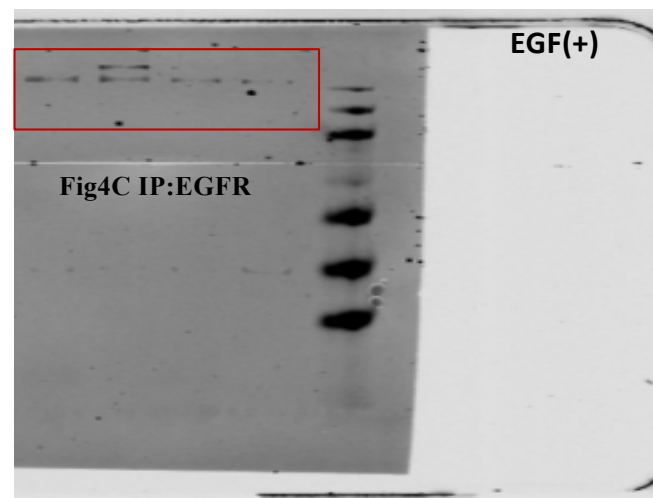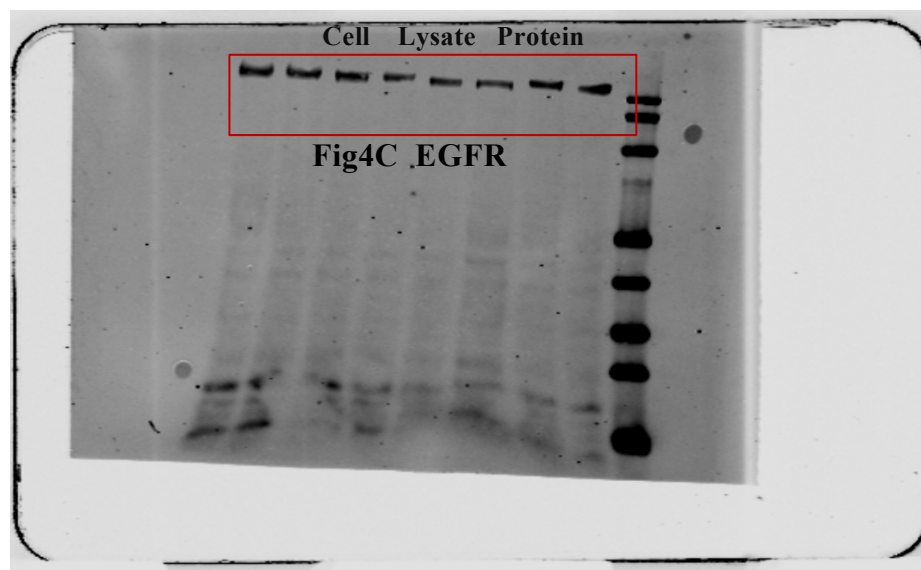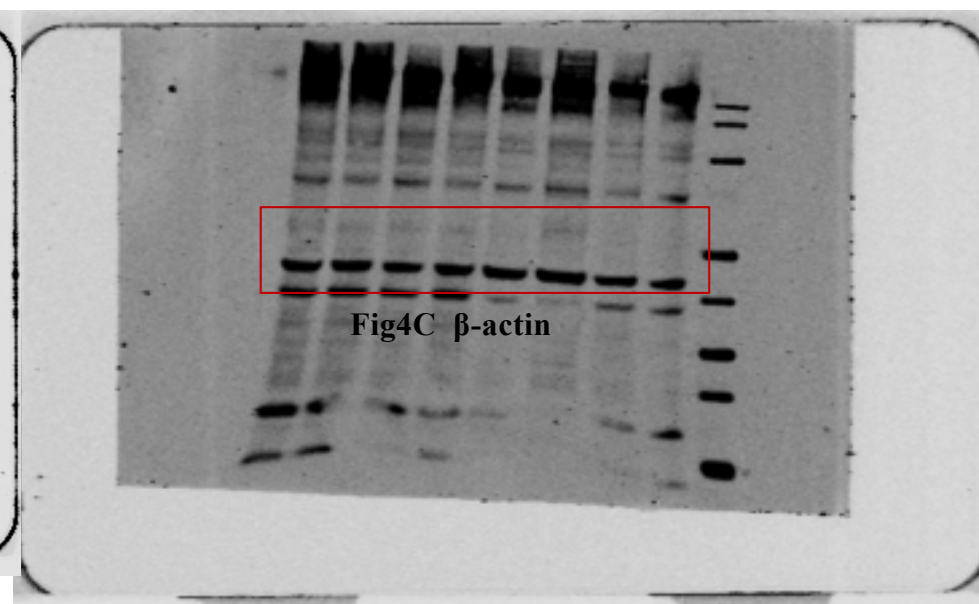

## Internalized EGFR

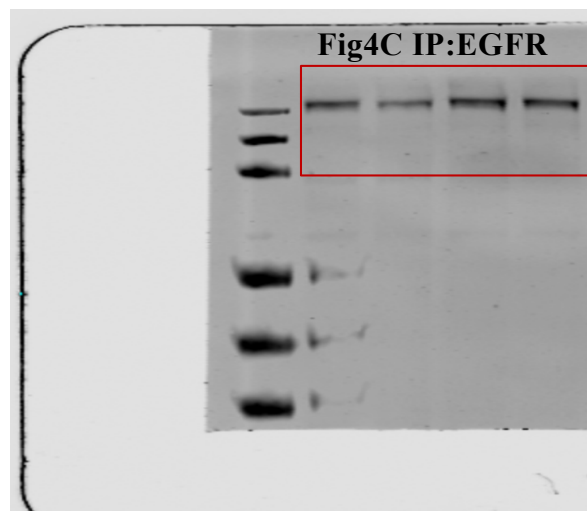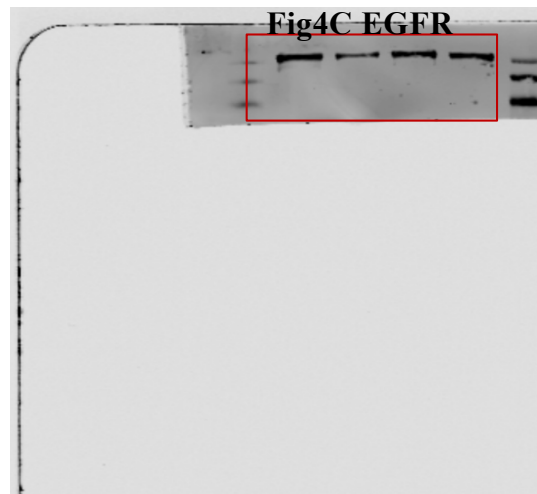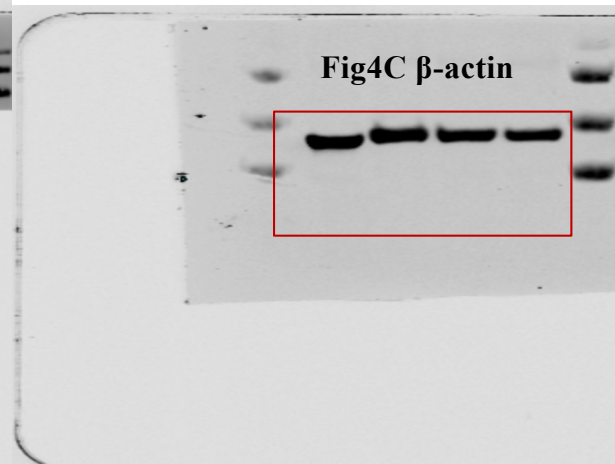

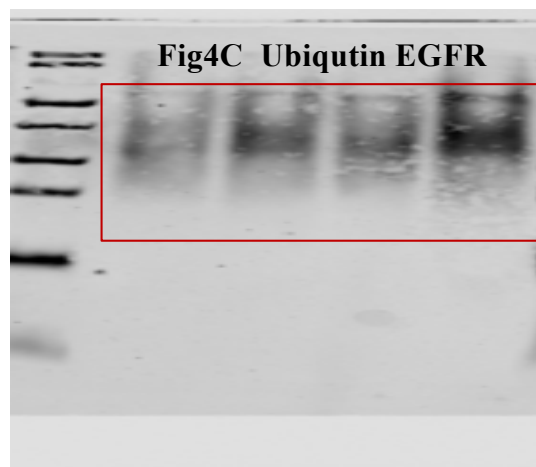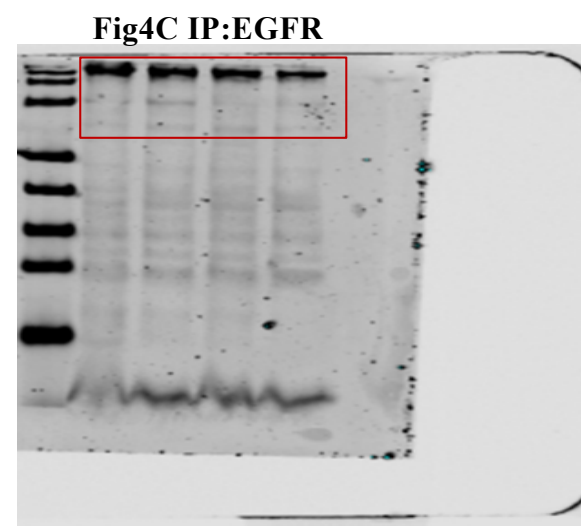

Supplement: S1 File — (PDF) [file pone.0231466.s001.pdf]
